# Deep-Water Fish Are Potential Vectors of Ciguatera Poisoning in the Gambier Islands, French Polynesia

**DOI:** 10.3390/md19110644

**Published:** 2021-11-17

**Authors:** Hélène Taiana Darius, Taina Revel, Philippe Cruchet, Jérôme Viallon, Clémence Mahana iti Gatti, Manoëlla Sibat, Philipp Hess, Mireille Chinain

**Affiliations:** 1 Institut Louis Malardé (ILM), Laboratory of Marine Biotoxins, UMR 241-EIO (IFREMER, ILM, IRD, University of French Polynesia), P.O. Box 30, 98713 Papeete, Tahiti, French Polynesia; trevel@ilm.pf (T.R.); pcruchet@ilm.pf (P.C.); jviallon@ilm.pf (J.V.); cgatti@ilm.pf (C.M.i.G.); mchinain@ilm.pf (M.C.); 2 Ifremer, DYNECO, Laboratoire Phycotoxines, F-44000 Nantes, France; manoella.sibat@ifremer.fr (M.S.); philipp.hess@ifremer.fr (P.H.)

**Keywords:** deep-water fish, ciguatera poisoning, rRBA, MBA, CBA-N2a, LC–MS/MS, composite ciguatoxicity, CTX1B analogs, Gambier Islands

## Abstract

Ciguatera poisoning (CP) cases linked to the consumption of deep-water fish occurred in 2003 in the Gambier Islands (French Polynesia). In 2004, on the request of two local fishermen, the presence of ciguatoxins (CTXs) was examined in part of their fish catches, i.e., 22 specimens representing five deep-water fish species. Using the radioactive receptor binding assay (rRBA) and mouse bioassay (MBA), significant CTX levels were detected in seven deep-water specimens in Lutjanidae, Serranidae, and Bramidae families. Following additional purification steps on the remaining liposoluble fractions for 13 of these samples (kept at −20 °C), these latter were reanalyzed in 2018 with improved protocols of the neuroblastoma cell-based assay (CBA-N2a) and liquid chromatography tandem mass spectrometry (LC–MS/MS). Using the CBA-N2a, the highest CTX-like content found in a specimen of *Eumegistus illustris* (Bramidae) was 2.94 ± 0.27 µg CTX1B eq. kg^−1^. Its toxin profile consisted of 52-*epi*-54-deoxyCTX1B, CTX1B, and 54-deoxyCTX1B, as assessed by LC–MS/MS. This is the first study demonstrating that deep-water fish are potential ciguatera vectors and highlighting the importance of a systematic monitoring of CTXs in all exploited fish species, especially in ciguatera hotspots, including deep-water fish, which constitute a significant portion of the commercial deep-sea fisheries in many Asian–Pacific countries.

## 1. Introduction

Throughout the tropical and subtropical Indo–Pacific, deep-water fish reside near the seafloor of oceanic islands and continental habitats, on the drop-offs of deep slopes and seamounts, and sometimes at depths of up to 1500 m [1,2,3]. Most species fished for human consumption belong to the Epinephelidae (grouper), Lethrinidae (emperor), and Lutjanidae (snapper) families [2,3,4,5,6,7]. These large carnivores are characterized by slow growth, delayed maturity, extended longevity, and high site fidelity, making them vulnerable to fishing pressure [4,8]. Highly prized as food in many regions, these deep-water fish are a valuable source of protein for indigenous communities [7,9]. The deep-water tropical demersal fisheries are small-scale subsistence fisheries but also support cultural, recreational, and commercial fisheries [2,3,8,9]. The main fishing techniques used to catch these fish are horizontal longlines, suspended or set on the bottom; vertical longlines; and manual, hydraulic, or electric reels [1,2]. In the snappers belonging to Etelinae subfamily (Lutjanidae family), the genera *Aphareus*, *Aprion*, *Etelis,* and *Pristipomoides* represent the main targeted species and are also commercially important marine species in many tropical–subtropical fisheries as well [4,9]. It is noteworthy that they account for a large proportion of catches of the Hawaiian (Deep Seven Bottom fish complex) and Japanese deep-water fisheries [2,3,4,5,6,7,10,11]. In French Polynesia, about 100 species have been identified, mainly snappers and groupers, the abundance of which and specific biodiversity vary from one archipelago to another, and for which 84 to 99% of the fish caught are sold on Tahitian markets [1].

In 2003, two professional fishermen from the island of Mangareva, in the Gambier Archipelago (French Polynesia), received complaints from customers who reportedly suffered from ciguatera poisoning (CP) following the consumption of “paru” (Tahitian generic name for deep-water fish). Owing to their deep-sea habitats, “paru” are not usually regarded as common CP vectors. However, earlier in the same year, a “paru” and an “uravena” (oilfish) were implicated in two toxic incidents reported from the Gambier Archipelago (Table S1). Ciguatera is classically defined as an ichthyosarcotoxism, i.e., a foodborne poisoning caused by the consumption of tropical reef fishes contaminated by ciguatoxins (CTXs) [12]. Historically, the first ciguatera toxin analog was isolated from the moray eel *Gymnothorax javanicus* by Scheuer et al. [13] and named “ciguatoxin”. Later, this same compound was structurally characterized by Murata et al. [14,15] and coded “CTX1B” by Satake et al. [16]. These compounds are potent polyether neurotoxins produced by benthic dinoflagellates in the genera *Gambierdiscus* and *Fukuyoa* that are generally found attached to macroalgal hosts [17]. Grazing of *Gambierdiscus*/*Fukuyoa* cells by herbivorous fish [18,19], which in turn are predated on by carnivorous fish, leads to the progressive biomagnification and biotransformation of CTXs along the trophic web, and ultimately to poisoning events in humans [20]. This complex disease is characterized by a myriad of symptoms, including gastrointestinal, neurological, cardiovascular, and general disorders, which may vary from one patient to another [21,22,23]. According to Halstead [24], more than 400 coral reef fish species are believed to be potential vectors of CP. Tropical areas surrounded by coral reefs such as in the Pacific, the Caribbean, and Indian Oceans are regarded as CP-endemic regions [25]. In addition to the fact that high incidence rates are consistently reported from these areas, causing significant health issues, CP medical management is complexified by the lack of (i) human diagnosis biomarkers, (ii) no proven effective antidote, and (iii) frequent occurrence of debilitating chronic manifestations [21,22,26]. Moreover, they also have detrimental effects on the economies of these island nations whose populations strongly rely on seafood consumption for their subsistence [23,25]. Since the early 2000s, this neglected tropical disease has also spread to several temperate-like regions/countries in Macaronesia and east and southeast Asia, possibly related to climate change [25]. Across the Pacific islands, traditional knowledge, handed down from generation to generation, has enabled local communities to develop not only folk remedies to treat ciguatera [27,28,29,30] but also avoidance strategies to keep the levels of poisoning as low as possible, e.g., thanks to a good knowledge of their environment and the help of homemade tests to detect ciguatoxic fish [31,32]. In addition, even in well-equipped laboratories, the detection of CTXs in seafood proves challenging [33,34,35], since CTXs are generally present at ultratrace concentrations ranging from pico- to nanograms per kilograms. They are odorless, colorless, and are not affected by cooking or freezing [20]. Indeed, the greatest challenge in CTX detection, regardless of the detection method used, is to be able to reach a sensitivity in accordance with the very low safety levels recommended by the US Food and Drug Administration (US FDA) for the Pacific and Caribbean CTXs, i.e., 0.01 µg CTX1B kg^−1^ and 0.1 µg C-CTX1 kg^−1^, respectively [36]. In this context, various methods have been developed to detect, identify, and quantify CTXs in seafood, including in vivo animal assays, cell-based assays, receptor binding assays, antibody-based immunoassays, electrochemical biosensors, and chemical analytical methods [37,38,39,40].

This study reports the ciguatoxin analyses performed on a batch of deep-water fish at the request of two local commercial fishermen, consisting of 22 specimens representing five deep-water fish species caught using traditional fishing techniques, i.e., longline reel fishing, outside of the Gambier barrier reefs at depths ranging from 100 to 300 m between October and December 2003. First, samples were extracted and analyzed for the presence of CTXs by the radioactive receptor binding assay (rRBA) and the mouse biological assay (MBA). These analyses, which were performed in 2004, revealed the presence of significant levels of CTX-like compounds in seven specimens distributed among all five deep-water species. In 2018, the remaining liposoluble fractions of several of these specimens (stored at −20 °C) were reanalyzed using improved extraction protocols and more sensitive and confirmatory detection methods, namely, the neuroblastoma cell-based assay (CBA-N2a) and the liquid chromatography tandem mass spectrometry (LC–MS/MS). Our findings allowed the confirmation of CTX contamination in several of these samples by providing additional information on the CTX analogs involved. Results of the comparison of toxicity data obtained by means of four detection methods are further discussed. The implication of the presence of CTXs in these high-value fish products in terms of ciguatera risk management is also addressed.

## 2. Results and Discussion

### 2.1. Ciguatoxin-like Activity as Assessed by MBA, rRBA, and CBA-N2a

Using the MBA, four specimens and five pooled samples belonging to the golden grouper *Saloptia powelli* (*n* = 15/15), the reticulate grouper *Epinephelus tuamotuensis* (*n* = 1/2), and the Crimson jobfish *Pristipomoides filamentosus* (*n* = 1/2) were found negative (Table 1). Only five specimens (#17, #18, #19, #20, and #22) belonging to *E. tuamotuensis* (*n* = 1/2), the deep-water longtail red snapper *Etelis coruscans* (*n* = 2/2), *P. filamentosus* (*n* = 1/2), and the brilliant pomfret *Eumegistus illustris* (*n* = 1/1), elicited ciguateric symptoms in mice, the most common being diarrhea, dyspnea, and ataxia as described in Lewis and Endean [41] (Table 1). The specimen #22, *E. illustris,* appeared as the most toxic specimen since the injection of a fraction of only 5 g flesh eq. of this fish was sufficient to induce death in tested mice, while the four other toxic fish required 10, 25, or 50 and up to 200 g flesh eq. to be lethal (Table 1). The total flesh toxicity values ranging from 0.005 to 0.2 mouse unit per gram of fish flesh (MU g^−1^), which can be converted to 0.04 to 1.4 µg CTX1B eq. kg^−1^ fish flesh, showed comparable toxicities as reported in coral reef carnivorous fish collected in the Asia-Pacific region [42,43,44,45].

**Figure 1:**
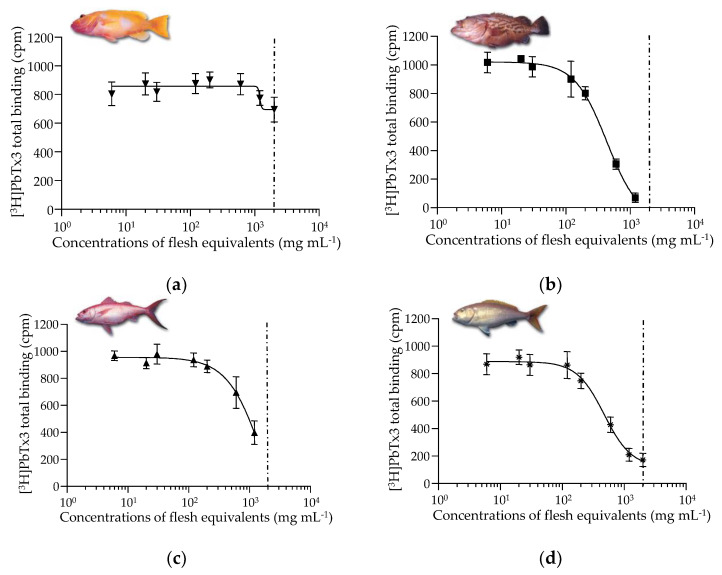

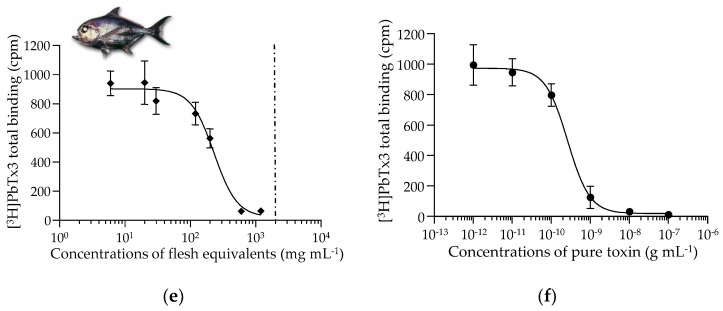
Binding dose-response curves of CTX1B and LF90/10 extracts of deep-water fish collected from the Gambier Archipelago. (**a**) *Saloptia powelli* #10; (**b**) *Epinephelus tuamotuensis* #17; (**c**) *Etelis coruscans* #18; (**d**) *Pristipomoides filamentosus* #20; (**e**) *Eumegistus illustris* #22; (**f**) CTX1B. Data represent the mean ± SD of three subsamples (5 g each) per specimen tested each in one experiment (*n* = 3) and CTX1B tested in three independent experiments (*n* = 3), each concentration run in duplicates. The dotted vertical line corresponds to the maximum concentration of extract in flesh tissue equivalent (MCE = 2000 mg flesh eq. mL^−1^) for matrix interference. The mean IC_50_ value obtained for CTX1B was 0.26 ± 0.07 ng mL^−1^ (*n* = 3) with a mean CV of 2.8%.

Using the rRBA, most of the fish samples (*n* = 15/22) showed no affinity toward site 5 of the voltage-gated sodium channels (VGSCs) (example Figure 1a), whereas seven specimens (examples Figure 1b–e) showed a response typical of the mode of action of VGSC activators such as CTX1B (Figure 1f) [46,47]. As illustrated in Figure 1, partial ((c): #18 and (d): #20) or full sigmoidal dose-response curves ((b): #17 and (e): #22) corresponding to low and high composite binding affinities, respectively, were obtained below the maximum concentration of extract that does not induce unspecific effect (MCE).

In the Serranidae family, most of the specimens of *S. powelli* (*n* = 14/15) tested negative, except for one specimen showing composite binding affinity, such as one specimen of *E. tuamotuensis* (Table 2).

Conversely, in the Lutjanidae and Bramidae, all specimens displayed composite binding affinities (Table 2). For all positive fish samples, the mean IC_50_ values ranged from 231 to 1260 mg flesh eq. mL^−1^, or 1.15 to 0.21 µg CTX1B eq. kg^−1^, respectively, after conversion (see Section 3.4.1). These values corresponded to the CTX-like contents found in the most potent *E. illustris* specimen #22 and the least potent *S. powelli* specimen #9, respectively, with coefficients of variation (CVs) ranging from 2.7 to 28.6% (Table 2). In French Polynesia, CTX-like contents up to 6 µg CTX3C eq. kg^−1^ (2.62 µg CTX1B eq. kg^−1^ by conversion) estimated by rRBA have also been reported in Serranidae and Lutjanidae [32,48,49,50]. Using the rRBA, the lowest CTX content found in fish remnants involved in ciguatera cases from French Polynesia was 0.16 µg CTX1B eq. kg^−1^ (clinical symptoms not available) [51]. Additionally, consumption of a toxic *Acanthurus xanthopterus* specimen estimated at 0.24 µg CTX1B eq. kg^−1^ was associated with gastrointestinal, neurological, muscular, and articular clinical manifestations in another patient [51]. Thus, the seven positive deep-water specimens would be unsafe for consumption.

The remaining liposoluble fractions of 13 out of 22 samples were submitted to additional purification steps, and the composite cytotoxic activity in LF90/10 and LF100 fractions was investigated by CBA-N2a. Results of the qualitative screening were negative (i.e., percentage of N2a cell viability above 80%) for four individual specimens (#6, #7, #8, #9) and one pooled samples (#10–11), all belonging to *S. powelli* (Table 3). Conversely, four other specimens (#17, #18, #20, #22) and one pooled samples (#1–3) showed specific activation of VGSCs in LF90/10 and/or LF100 extracts inducing a reduction in cell viability >20% (decision limit defined in Viallon, et al. [52]) only in OV+ condition. Therefore, they were further tested at eight distinct concentrations (quantitative mode) (Table 3).

**Figure 2:**
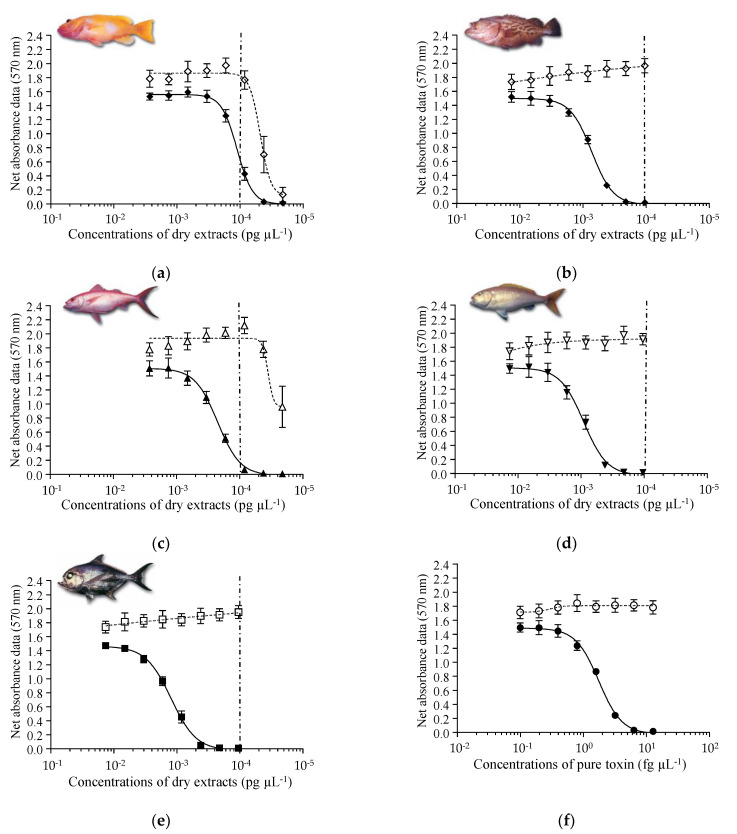
Dose-response curves of N2a cells in OV− (open symbols) and OV+ (solid symbols) conditions exposed to increasing concentrations of CTX1B or LF90/10 dry extracts of deep-water fish collected from the Gambier Archipelago. (**a**) *Saloptia powelli* #1–3; (**b**) *Epinephelus tuamotuensis* #17; (**c**) *Etelis coruscans* #18; (**d**) *Pristipomoides filamentosus* #20; (**e**) *Eumegistus illustris* #22; (**f**) CTX1B. Data represent the mean ± SD of four independent experiments, each concentration run in triplicate. The dotted vertical line corresponds to the maximum concentration of LF90/10 dry extracts (MCE = 10,000 pg µL^−1^) for matrix interference. The mean EC_50_ value obtained for CTX1B was 1.76 ± 0.07 pg mL^−1^ (*n* = 4) with a CV of 4%.

When exposed to increasing concentrations of these fish extracts, N2a cells displayed a typical sigmoidal dose-response curve under OV+ condition (Figure 2a–e), whereas no cytotoxic activity was observed in OV− condition (Figure 2a–e), a pattern typical of the mode of action of VGSC activators such as CTX1B (Figure 2f) [52,54,55].

For all fractions found positive by CBA-N2a, the raw EC_50_ values expressed in pg µL^−1^ of dry extract were further converted to mg flesh eq. mL^−1^, according to “dry extract weight/fresh flesh weight” ratio (Table 3 and Section 3.4.3). The mean EC_50_ values ranged from 0.63 to 17.92 mg flesh eq. mL^−1^, showing good repeatability, with CVs varying from 6.5 to 13.3% (Table 3). The deep-water fish samples showing the highest composite cytotoxic activity were, in decreasing order: *E. illustris* #22, *E. tuamotuensis* #17, *E. coruscans* #18, and *P. filamentosus* #20, for which complete sigmoidal dose-response curves were obtained below the MCE (matrix effect) limit (Figure 2b,d,e). The CTX-like contents were between 0.10 and 2.83 µg CTX1B eq. kg^−1^, i.e., from the least potent *S. powelli* pooled samples #1–3 to the most potent *E. illustris* specimen #22. Among all fish samples, *E. illustris* #22 was the most toxic fish with a total CTX-like content of 2.94 ± 0.27 µg CTX1B eq. kg^−1^ as estimated by CBA-N2a (LF90/10+LF100).

### 2.2. Ciguatoxin Profiles as Assessed by LC–MS/MS

**Figure 3:**
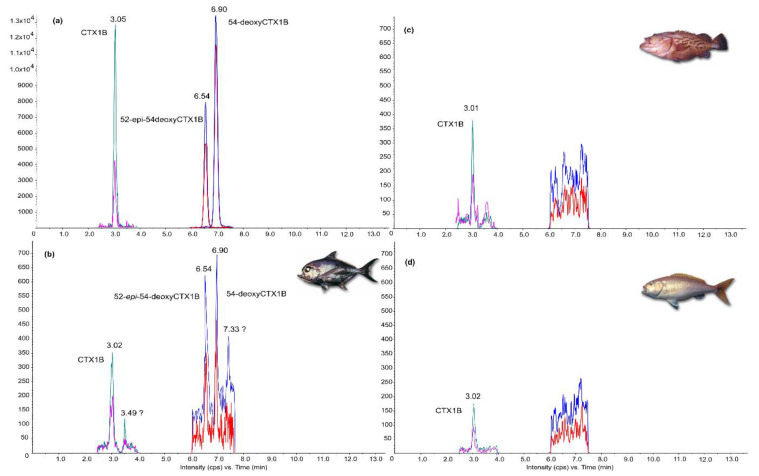
Liquid chromatography tandem mass spectrometry (LC–MS/MS) chromatograms acquired in positive scheduled multireaction monitoring (MRM) mode (Section 3.5). (**a**) A mix of Pacific CTX standards CTX1B at 3.10 min, 52-*epi*-54-deoxyCTX1B at 6.54 min, and 54-deoxyCTX1B at 6.94 min. (**b**) Chromatogram of *Eumegistus illustris* specimen #22. (**c**) Chromatogram of *Epinephelus tuamotuensis* specimen #17. (**d**) Chromatogram of *Pristipomoides filamentosus* specimen #20. The *m/z* transitions correspond to 1128.6/1075.6 (green); 1128.6/95.1 (pink); 1112.6/1077.6 (blue); and 1112.6/1059.6 (red).

Among the remaining liposoluble fractions of 13 out of 22 samples, only 10 of them could have been submitted to additional purification steps adapted for the LC–MS/MS analyses. The presence of CTX1B analogs was confirmed in three deep-water fish samples: *E. tuamotuensis* #17, *P. filamentosus* #20, and *E. illustris* #22 (Figure 3 and Table 4).

The quantification assessed by LC–MS/MS ranged from 0.08 to 1.11 µg CTX1B kg^−1^, *E. illustris* #22 still being the most potent deep-water fish. Only CTX1B was detected in *E. tuamotuensis* #17 and *P. filamentosus* #20, while the *E. illustris* specimen #22 exhibited the more complete toxin profile with three known CTX analogs in decreasing order: 52-*epi*-54-deoxyCTX1B, CTX1B, and 54-deoxyCTX1B (Figure 3, Table 4). Its toxin profile also included a putative analog of CTX1B showing a peak at 3.49 min, which corresponds to the *m/z* 1128.6/1075.6, but could not be identified by the other *m/z* transitions, as the peak intensity was too low (Figure 3b). A second putative CTX analog showing a peak at 7.33 min, which corresponds to the *m/z* 1112.6/1077.6, could not be identified either (Figure 3b). Thus, neither of these two putative unknown CTX analogs could be considered in the final estimate of the CTX content of *E. illustris #22* (Table 4).

**Figure 4:**
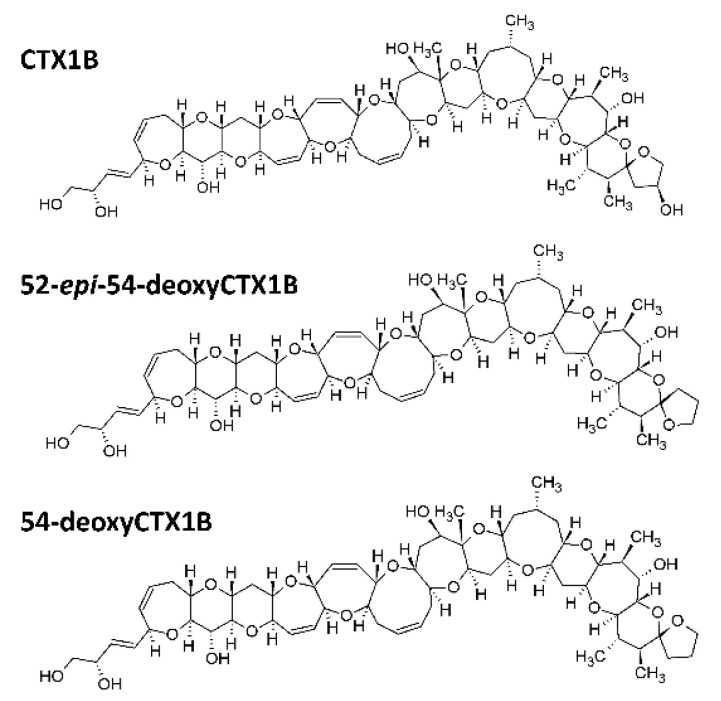
Chemical structures of CTX1B, 52-*epi*-54-deoxyCTX1B, and 54-deoxyCTX1B.

In contrast, CTX3C, 49-*epi*CTX3C, CTX4A, and CTX4B were not detected in any of the deep-water fish samples analyzed. Of note, 52-*epi*-54-deoxyCTX1B and 54-deoxyCTX1B were originally given the names CTX-2 and CTX-3, respectively [46]. For the record, CTX1B and the two deoxy analogs of CTX1B (Figure 4), 52-*epi*-54-deoxyCTX1B and 54-deoxyCTX1B, are usually found in a variety of great carnivores such as moray eels, barracudas, groupers, snappers, Spanish mackerels, or jacks in the Asia–Pacific region [43,44,45,46,51,56,57,58,59,60,61,62,63,64]. However, these analogs were also reported in herbivorous and omnivorous coral reef fish species [53,61]. Of note, these results highlight the remarkable stability of CTXs over time, since the two series of analyses described herein were performed 15 years apart.

Establishing a risk map of fishing sites around the Gambier Archipelago appears difficult given the limited number of specimens analyzed per site. However, Tenoko seemed to be at lower risk than Tokorua where all the fish collected (#17, #19, #21, #22) were found positive, with at least one of the four detection methods displaying high CTX levels (Table 1, Table 2, Table 3 and Table 4).

### 2.3. Comparisons of CTX-like Contents in Deep-Water Fish Using Four Detection Methods

The toxic status of all 22 specimens as determined by MBA, rRBA, CBA-N2a, and LC–MS/MS is summarized in Table 5. Fish samples were considered negative if all detection methods gave negative results versus positive when at least one detection method gave positive results.

When MBA and rRBA results were compared (Table 5), no symptom was observed in mice for any of the 15 fish classified as negative by rRBA whereas typical ciguatera symptoms were reported in mice for 5 of the 7 fish found positive using the rRBA (Table 1 and Table 2). Despite a LOD of 0.035 μg CTX1B eq. kg^−1^, the MBA failed to detect CTXs in #9 and #21, while levels of 0.21 and 0.55 μg CTX1B eq. kg^−1^ were measured by the rRBA. The different results obtained between these two methods could be explained by (i) the use of different extraction protocols with different CTX recovery percentages, (ii) insufficient amount of flesh tested in the MBA to detect this level of CTXs, and (iii) the composite binding affinity detected by rRBA in both samples that does not seem to be related to a composite toxicity in mice. It should be noted that 52-*epi*-54-deoxyCTX1B is twice as potent as CTX1B to bind to VGSC receptors but twice as less toxic in mice, as highlighted by Yogi et al. [62]. Although the identity and proportion of CTXs present in these two samples are unknown, this difference does not seem inconsistent when considering all these assumptions. Overall, these data suggest a good agreement between MBA and rRBA for 20 out of 22 samples (90%), a result consistent with previous observations for the detection of CTXs [65].

When MBA and CBA-N2a data were compared for 13 fish (Table 5), six specimens were found negative by both methods (Table 1 and Table 3). Among the four specimens found positive by both methods, the CBA-N2a consistently gave higher CTX values (Table 5). The only discrepancy observed between MBA and CBA-N2a data concern the pooled samples of three *S. powelli* #1–3 in which traces of CTXs were detected by CBA-N2a. Overall, a good agreement was obtained between these two methods for 10 out of 13 samples (77%).

When comparing rRBA and CBA-N2a data for 13 fish samples, the nontoxic and toxic statuses were validated by both methods for 69% of samples (i.e., 9/13 fish) (Table 5). Regarding the CTX-like contents in the four positive fish, significant differences were observed between *E. tuamotuensis* #17, *E coruscans* #18, and *E. illustris* #22 (*p* < 0.05) with CBA-N2a yielding CTX content values two to four times higher than those of rRBA, whereas no significant difference (*p* > 0.05) was observed for *P. filamentosus* #20 (Table 5). Interestingly, our results contradict those from previous studies, which showed an overestimation by rRBA compared to CBA-N2a. It is worth mentioning that the potency of CTX analogs as assessed by rRBA and CBA-N2a does not always correlate when purified CTXs are tested individually [47]. For example, 52-*epi*-54-deoxyCTX1B (ex CTX2A2) showed higher binding affinity (rRBA) but lower cytotoxicity on N2a cells than 2,3-dihydro-2,3-dihydroxyCTX3C (ex CTX2A1) [47]. Thus, knowing that fish actually harbor a mixture of CTX analogs in variable proportions, the combined effects of all CTX analogs could induce variable affinity and cytotoxicity, explaining, in part, the differences observed between methods for some of our samples. It remains to be clarified whether this difference could also be due to the different extraction protocols used [53,54,66,67,68].

When comparing the CBA-N2a results with those obtained by the two other biological assays (MBA and rRBA), the nontoxic and toxic statuses were confirmed for 9/13 samples, showing a 69% agreement between these three methods (Table 5). Slight discrepancies were found for only one pooled sample and one specimen contaminated with low levels of CTXs: (i) CBA-N2a detected the presence of 0.10 µg CTX1B eq. kg^−1^ in a pooled sample of *S. powelli* (#1–3), which was found negative by rRBA and MBA, a result consistent with the higher sensitivity of the CBA-N2a, as compared to MBA and rRBA [35,53,54,66,67,68], and therefore is more likely to detect traces of CTXs); (ii) *S. powelli* #9, which showed the lowest IC_50_ value as measured by rRBA, turned out negative by both MBA and CBA-N2a. Again, this result is not surprising since it is well established that CTXs with a high affinity for sodium channels do not necessarily display a high cytotoxic activity and/or acute toxicity in mice [47,62].

Regarding CTX contents estimated by LC–MS/MS, they were like those obtained by rRBA and MBA for *E. illustris* #22 (Table 5). In two other positive deep-water fishes, LC–MS/MS gave CTX estimates two to nine times lower than those of rRBA and CBA-N2a for *E. tuamotuensis* #17 and *P. filamentosus* #20 for which only one CTX1B analog was detected. Additionally, LC–MS/MS did not detect CTXs in the pooled samples #1–3 of *S. powelli* and *E. coruscans* #18, which were found positive with CBA-N2a in the case of *S. powelli* and by all three biological methods in the case of *E. coruscans* (Table 5). The fact that LC–MS/MS analysis consistently resulted in lower results than any other method could be explained by (i) matrix effects and low recovery from additional sample preparation with cartridge column, (ii) the additional presence of several other known analogs below the detection limit, (iii) the presence of additional hitherto undescribed analogs, (iv) the inaccuracy of the equal-response assumption or the toxic equivalency factors, or (v) a combination of the above factors. Overall, the nontoxic (one specimen and one pooled sample) and toxic (three specimens) statuses were confirmed by LC–MS/MS for 6/10 samples showing a 60% agreement between the three biological and the chemical methods. Of note, such discrepancies were also observed between CBA-N2a and LC–MS/MS data in fish from the Selvagens Islands, suggesting the presence of metabolic products, which need to be further elucidated, as well [69]. In this region, C-CTX1 was the most frequently encountered Caribbean CTX analog, although the toxin profile of the zebra bream *Diplodus cervinus* was free of it [69]. Similarly, our results show that CTX1B was not the main contributor to the toxicity of the *E. illustris* specimen from the Gambier Islands. Our results also suggest that the other deep-water fish species analyzed in this study may contain metabolized Pacific CTX analogs that differ from the ones typically metabolized by lagoon coral reef fish. Nevertheless, the limited number of specimens analyzed per species due to opportunistic sampling requires further work to provide a better estimate of CTX analogues incidence in these species.

From a technical point of view, the divergences observed on a small number of specimens between MBA, rRBA, CBA-N2a, and LC–MS/MS results could also be explained by the use of different CTX extraction protocols in our study. Indeed, CTX recovery in fish flesh can vary from 23 to 90% depending on the matrix complexity and the extraction protocol used [70,71,72]. Therefore, the utilization of different combinations of extraction protocols and detection methods likely impact the final CTX quantifications in fish. Additionally, the presence of multiple CTX analogs in biological matrices presenting varying affinities to sodium channels could well result in varying degrees of cytotoxic activity and/or acute toxicity due to the differences in CTX analogs’ potencies [46,47,48,62,67,73,74]. Among the three CTX1B analogs identified by LC–MS/MS in *E. illustris* #22, CTX1B is known to be highly potent, as it, alone, could contribute to 55 to 92%, on average, of the total ciguatoxicity in the blue spotted grouper *Cephalopholis argus* [60].

### 2.4. Contribution of Deep-Water Fish to the Trophic Chain of Ciguatera

Despite the low number of specimens analyzed per species and per site in this study and the limitation inherent to the use of different extraction protocols, evidence for the presence of CTXs in several of the deep-water fish species at levels 4 to 282 times higher than the guidance level of 0.01 µg CTX1B kg^−1^ recommended by the US FDA and EFSA [36,75] and above 0.16 µg CTX1B eq. kg^−1^ found in fish remnants in French Polynesia [51] highlights the potential health risk associated with the consumption of these fish in French Polynesia.

Deep-water fish species are not commonly regarded as CP vectors, as these organisms usually reside below 100 m to 1500 m [1,2,3]. Although cells of *Gambierdiscus*, the causative agent of ciguatera, have been occasionally reported at depths between 30–50 m [76,77,78,79], this finding that “paru” can harbor significant levels of ciguatoxins is more likely linked to the feeding habits of these deep-water species that show strong diel vertical migration behavior, swimming into the water column at night, at depths consistent with feeding on mesopelagic boundary community micronekton such as finfishes, squids, shrimps, crabs, amphipods, ascidians, myctophids, and ophichthid eels [2,80,81]. Coral reef fish belonging to various trophic levels (i.e., herbivorous, omnivorous, and carnivorous species), which represent potential prey organisms for these deep-water predators, are allegedly CTX reservoirs [21]. In the same way, marine invertebrates and crustaceans such as octopus and crabs are also likely potential carriers of CTXs [54,61,82]. As all these organisms are commonly found within the lagoons, around the pass, and outside the barrier reefs, CTX flux between these different habitats may have possible connections to deep-sea habitats when their inhabitants migrate vertically to upper seawater layers to feed.

Three distinct CTX analogs were formally characterized in the toxic fish specimens, namely CTX1B and two deoxy analogs of CTX1B, i.e., 52-*epi*-54-deoxyCTX1B and 54-deoxyCTX1B. Interestingly, these compounds are believed to derive from the biotransformation (i.e., enzymatic oxidation) of algal precursors such as CTX4A and CTX4B [83]. Although no *Gambierdiscus* data were collected at the time of fish sampling, the marked abundance of *Gambierdiscus* populations within the lagoon of the Gambier Islands—cell estimates between 400,000 and 1,000,000 cells g^−1^ wet weight algae—were reported from this area [84,85]. This suggests *Gambierdiscus* is the primary source of these CTXs, which are further transferred and transformed through the trophic chain to the deep-water fish specimens analyzed in the present study. Indeed, the Gambier area is recognized as a biodiversity “hotspot” of *Gambierdiscus*, with up to six *Gambierdiscus* species found to coexist within a single sampling site in Rikitea Bay (Mangareva Island) [86,87]. Toxicological investigations conducted on in vitro cultures of two strains of *G. polynesiensis* isolated from benthic assemblages collected from Rikitea Bay further revealed a high toxin production (around 3.3 ± 0.2 pg CTX3C eq. cell^−1^) in one isolate (RIK7) [88]. A remarkably diverse toxin profile was evidenced by the formal identification of seven distinct CTX analogs in cultured cells, including CTX3B, CTX3C, CTX4A, and CTX4B [88,89]. These findings confirmed *G. polynesiensis* as a key toxin producing species in the South Pacific [17,51]. Finally, field samplings conducted between 2012 and 2013 in various study sites within Mangareva Lagoon also showed 45 to 73% of coral reef fish, including both herbivorous and carnivorous fish species, were found positive to CTXs by CBA-N2a (data not shown). All these observations, which support the high CP incidence rates consistently reported from the Gambier Islands since the 1960s [90,91,92], suggest a very active flux of algal CTXs entering the marine food web in this area. Of note, the only other ciguatera case involving deep-water fish recorded in the Gambier Islands occurred in 2020 following the consumption of a *Ruvettus pretiosus* specimen causing typical gastrointestinal and neurological symptoms such as cold allodynia and tingling manifestations (Table S1). This species was previously mentioned to be at risk of ciguatera by Halstead et al. [93].

## 3. Materials and Methods

### 3.1. Study Site

**Figure 5:**
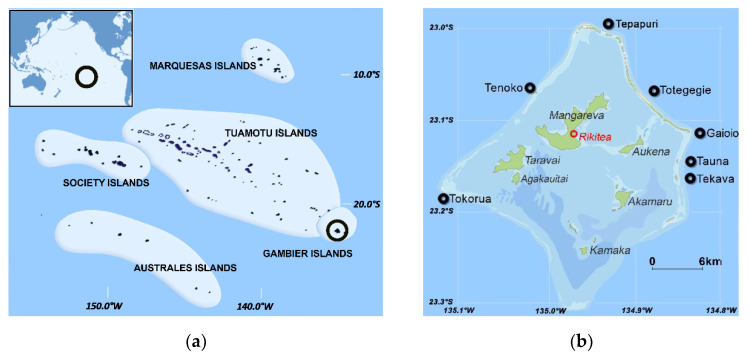
Maps of (**a**) French Polynesia and (**b**) Gambier Archipelago showing the geographic location of the seven fishing sites where fish samples were collected in 2003 by the two local fishermen at depths between 100–200 m offshore the coral reef barrier.

Located in the South Pacific Ocean, French Polynesia is composed of 118 islands grouped in five archipelagos, namely Society, Australes, Marquesas, Tuamotu, and Gambier, covering 4200 km^2^ of land area, but scattered over a surface of 2,500,000 km^2^ (Figure 5a). The Gambier Archipelago (23°07′04″ S–134°58′13″ W) is located approximately 1600 km Southeast of the main island of Tahiti, constituting the eastern end of French Polynesia. Covering an area of 30 km^2^, the Gambier Archipelago is composed of high islands of volcanic origin along with coral islets on the surrounding fringing reef (Figure 5b). The village of Rikitea is the capital of the main island Mangareva as well as of the Gambier Archipelago.

The Gambier Islands have been a well-known hotspot for CP for over five decades [90,94,95], with consistently high annual incidence rate (IR) reported annually, which varied from 35 to 801 cases/10,000 inhabitants between 2000 and 2006 (mean IR of 364 cases/10,000 inhabitants based on 2002 census, 1085 inhabitants; www.ispf.pf/bases/Recensements/2002.aspx, accessed on 29 April 2021), and from 46 to 569 cases/10,000 inhabitants between 2007 and 2018 (mean IR of 316 cases/10,000 inhabitants) [90,91,92]. Two CP events involving “uravena” and “paru” occurred in May and September 2003 in the Gambier Islands, but clinical details and fish species identification were unfortunately unknown (Table S1).

### 3.2. Fish Sample Collection

The 22 deep-water fish specimens analyzed in this study were provided by two local fishermen practicing longline reel fishing. Fish specimens were caught between October and December 2003 from seven different fishing sites located off the coral reef barrier at depths ranging from 200 to 350 m (Figure 5b). Once received at the Institut Louis Malardé (ILM), their fork length and weight were measured to the nearest centimeter and gram (Table S2). Each specimen was identified at the species level based on morphological characteristics as described in the report of Ponsonnet [1] and according to updated taxonomy of these deep-water species [96]: *Saloptia* (ex *Plectropomus) powelli* (*n* = 15), and *Epinephelus tuamotuensis* (*n* = 2) for the Serranidae; *Etelis coruscans* (*n* = 2) and *Pristipomoides filamentosus* (*n* = 2) for the Lutjanidae; and *Eumegistus illustris* (*n* = 1) for the Bramidae (Figure S1). The flesh of each fish was weighed, prepared separately in the form of filets, and stored at −20 °C until chemical extraction and analyses by biological or chemical methods.

### 3.3. Ciguatoxin Standard

A large stock of CTX1B standard was established at ILM since the 1990s, following the major purification work conducted on livers of moray eels using nuclear magnetic resonance (NMR) and fast-atom bombardment tandem mass, as described in previous studies [15,56,97]. The successive aliquots of CTX1B needed for the calibration of the different assays conducted in the present study (in 2003 and 2018) were prepared from this material as well as for previous studies, highlighting a stable activity of CTXs across the years [32,47,48,53,63,74,98,99,100,101,102,103].

For confirmatory LC–MS/MS analyses, the 52-*epi*-54-deoxyCTX1B and 54-deoxyCTX1B pure toxin standards were provided by the ILM and the Japan Food Research Laboratory (JFRL), respectively. To quantify the different CTX congeners and due to the lack of standards, concentrations were estimated from the calibration curves of CTX1B assuming equivalent molar response for other analogs.

### 3.4. Biological Methods

#### 3.4.1. Radioactive Receptor Binding Assay (rRBA)

**Figure 6:**
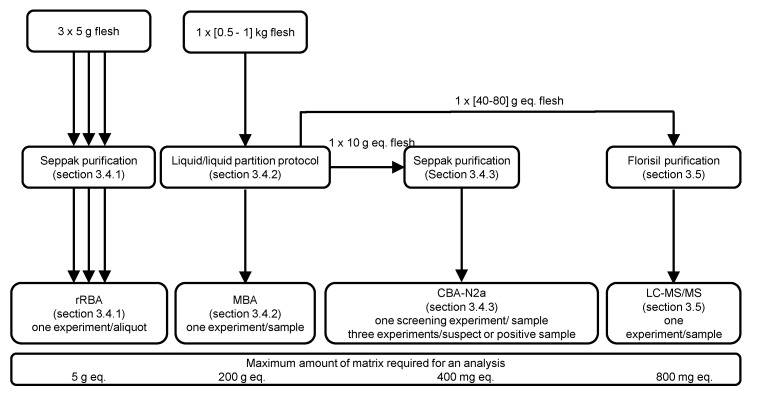
Summary of the analyses conducted on deep-water fish samples collected by two local commercial fishermen from the Gambier Archipelago. Analyses by the radioactive receptor binding assay (rRBA) and mouse biological assay (MBA) (*n* = 22) were carried out in 2003/2004 and, neuroblastoma cell-based assay (CBA-N2a) (*n* = 13/22) and liquid chromatography tandem mass spectrometry (LC–MS/MS) (*n* = 10/13) were performed in 2018.

For rRBA analyses, after homogenization of the whole flesh sample, CTXs were first extracted from three subsamples (5 g each) per specimen (Figure 6) following the protocol published later by Darius et al. [48].

Briefly, each subsample (5 g) was resuspended with 7 mL methanol (MeOH) in 50 mL tubes (Greiner Bio One, Frickenhausen, Germany) and sonicated for 2 h. After one night at −20 °C, the crude extracts were centrifuged and further purified on Sep-Pak C18 cartridges (360 mg sorbent per cartridge; Waters^®^, Saint-Quentin, France). The columns were preconditioned with aqueous methanol (MeOH/H_2_O) 70/30 (*v/v*) before loading extracts, 3 mL of H_2_O and 7ml of sample extract, were deposited in sequence on the top of the cartridge, which was then washed with 7 mL of MeOH/H_2_O 70/30 and successively eluted with 7 mL MeOH/H_2_O 90/10 and 7 mL of pure MeOH (100), resulting in three distinct liposoluble fractions (LF): LF70/30, LF90/10, and LF100. These fractions were then dried in a SpeedVac concentrator and resuspended in incubation buffer at a concentration of 5 g flesh equivalents (eq.) mL^−1^ and stored at 4 °C. As CTXs are mainly recovered in the LF90/10 fraction [53,74,102], only the LF90/10 fraction was considered to search for the presence of CTXs using the rRBA. This method is based on the competitive binding between CTXs and tritiated brevetoxins on site 5 of the α subunit of the VGSCs [104,105]. The rRBA analyses were performed in test tube format according to the protocol described by Darius et al. [48]. The brevetoxin PbTx-3 and [^3^H]PbTx-3 (15 Ci/mmol) were obtained from Latoxan (Rosans, France) and Amersham Perkin Elmer Life Science, respectively. The final assay concentration of [^3^H]PbTx-3 was 0.85 nM. The rat brain synaptosomes were prepared following the protocol described by Dechraoui et al. [47], with protein final assay concentration ranging from 60–80 µg mL^−1^ giving no more than 10% of the total radioactivity. The radioactivity was determined using a Perkin Elmer Microbeta Trilux 1450 liquid scintillation counter in 2 mL Perkin Elmer Betaplate scintillation cocktail. Nonspecific binding was measured in the presence of saturating concentration of PbTx-3 (0.67 µM) and subtracted from the total binding to yield specific binding. For the pure CTX toxin, six distinct concentrations (obtained by a serial dilution 1:10) of a CTX1B standard solution sourced from ILM ranging from 10^−12^ to 10^−7^ g mL^−1^ were tested in three independent experiments (*n* = 3). The concentration (Cx) of 2000 mg flesh eq. mL^−1^ corresponds to the maximum concentration of extract (MCE) that does not induce nonspecific binding on VGSCs from samples of *S. powelli* found negative by both rRBA and MBA. For deep-water fish samples, the three subsamples (5 g) per fish sample were tested in the same experiment at seven distinct concentrations ranging from 6 to 1200 mg mL^−1^ (final concentrations) of flesh tissue equivalent for half of the samples (i.e., #1 to #6, #17 to #19, #21, #22) and at eight concentrations ranging from 6 to 2000 mg mL^−1^ for the remaining half samples (i.e., #7 to #16, #20), each concentration tested in duplicate. The dose-response curves were established by plotting net count per minute (cpm) values vs. pure toxins or fish concentrations tested based on the four parameters logistic regression model (4PL) using GraphPad Prism software version 9.0.2. (GraphPad, San Diego, CA, USA). From these dose-response curves, the effective concentration, which causes 50% inhibition (IC_50_), was determined. For the pure CTX1B from the ILM, the mean IC_50_ value was 0.27 ± 0.01 ng mL^−1^ (*n* = 3). The limit of detection (LOD) and quantification (LOQ) for fish samples were estimated to 0.07 ± 0.02 and 0.13 ± 0.01 µg CTX1B eq. kg^−1^ of flesh, respectively, according to the following formula:

LOD = (IC_80_ of CTX1B/MCE) (1)

LOQ = (IC_50_ of CTX1B/MCE) (2)

The toxin content of fish extracts was estimated using the following equation and expressed in µg CTX1B eq. kg^−1^ of fish flesh:

Toxin content = (IC_50_ of CTX1B/IC_50_ of fish samples) (3)

The final toxin content was the mean ± standard deviation (SD) of 3 × 5 g flesh eq. per fish sample tested during the same rRBA experiment.

#### 3.4.2. Mouse Bioassay (MBA)

For the MBA, which requires larger amounts of biological material, sample extraction followed the protocol published later by Laurent et al. [106] with some slight modifications in the first step. Of note, as the flesh weight was too low for several specimens of the species *S. powelli*, specimens originating from the same fishing area were pooled (Table 1 and Table S2). Briefly, for each fish specimen, 1 kg of flesh (Figure 6) was extracted twice in 2 L acetone under sonication for 4 h. After one night at −20 °C, the resulting crude extracts were filtered and centrifuged for 10 min at 2800× *g*, then supernatants were pooled and dried under vacuum. The following steps were carried out identically to the extraction protocol previously described by Laurent et al. [106]. The resulting dried extract was further partitioned between 1 L dichloromethane (CH_2_Cl_2_) and 2 × 500 mL MeOH/H_2_O 60/40, shaken, and left at room temperature overnight. The resulting CH_2_Cl_2_ phase was dried under vacuum and further defatted by a second partition step using 2 × 200 mL cyclohexane and 100 mL MeOH/H_2_O 80/20. The liposoluble fraction (CH_2_Cl_2_ purified into MeOH/H_2_O 80/20) and the hydrosoluble fraction (MeOH/H_2_O 60/40) likely to contain CTXs and maitotoxins (MTXs), respectively, were then evaporated, dried under vacuum, and weighed. Upon completion of the MBA analyses, the remaining liposoluble extracts for two pooled samples and eight individual specimens (i.e., 13 out of the 22 tested specimens) were stored as archived fractions at −20 °C until 2018 and were further used in CBA-N2a and LC–MS/MS analyses.

The liposoluble extracts were tested for their toxicity using the MBA according to Legrand et al. [97]. Groups of two 18–21 g Swiss mice were intraperitoneally (i.p.) administered one dose of dried extract emulsioned in 250 µL of a 0.9% saline solution containing 0.1% Tween 60. Mice were observed over 24 h, and signs of intoxication and time of death were recorded and analyzed. First, fractions of 50, 100, and up to 200 g eq. flesh were tested when there was sufficient large amount of flesh. Fish samples were considered nontoxic if the injection of the maximal dose was not lethal. If fish were sufficiently toxic and enough amount of flesh equivalent remained, doses of 2.5, 5, and 10 g eq. flesh were also i.p. injected into each of two mice. Mice were sacrificed following animal protection protocols according to European Community Council directive 86/609/EEC. The total lethality of deep-water fish extracts was deduced from the mean survival time of injected mice, based on an in-house dose/survival time graph established experimentally in the laboratory. The lethal dose (LD_50_) is defined as the i.p. dose of fish flesh killing 50% of a group of two mice (18–21 g) over 24 h, and the lethal potency of CTX1B was 0.35 µg kg^−1^ [15,107]. One mouse unit (MU) is the LD_50_ for a 20 g mouse, which is equivalent to 7 ng of CTX1B [43]. Total fish flesh toxicity is expressed in mouse unit per gram of fish flesh (MU g^−1^) [43]. CTX-like content of deep-water fish was expressed in µg CTX1B eq. kg^−1^ of fish flesh. The limits of detection/quantification LOD/LOQ of the MBA was defined as 0.005 MU g^−1^ of fish flesh corresponding to 0.035 µg CTX1B eq. kg^−1^ of fish flesh.

#### 3.4.3. Neuroblastoma Cell-Based Assay (CBA-N2a)

For CBA-N2a analyses, an aliquot of the liposoluble fraction equivalent to 10 g of flesh was dried and resuspended in methanol for each of the 13 available deep-water fish samples (Figure 6). After purification on Sep-Pak C18 cartridges following the same protocol as the one used for rRBA samples, the LF90/10 and LF100 extracts were resuspended in dimethyl sulfoxide (DMSO) at a concentration of 10 mg mL^−1^ of dry extract and stored at −20 °C. The CBA-N2a analyses were conducted using mouse neuroblastoma (N2a) cell line (CCL-131) purchased from the American Type Culture Collection (ATCC, Manassas, VA, USA) and according to the optimized protocol published later by Viallon et al. [52]. The initial and final viabilities of the N2a cells were checked in the absence and presence of a nondestructive treatment of ouabain and veratridine (OV− and OV+ conditions, respectively) with O/V concentrations ranging from 80/8 to 100/10 µM. First, a qualitative screening approach was applied to LF90/10 and LF100 fractions of each fish (see supplementary materials of Viallon et al. [52]) by testing a single concentration (Cx) corresponding to approximately 10,000 ng mL^−1^ of dry extract (established as the maximum concentration of dry extract or MCE that does not induce nonspecific cytotoxic effects on N2a cells). The qualitative screening results of LF90/10 and LF100 extracts are expressed in N2a cell viability percentages (%) relative to their corresponding controls in OV− (COV−) and OV+ (COV+) conditions according to the following equations:

Viability percentage (%) = (net absorbance of Cx/net absorbance of COV−) × 100(4)

Viability percentage (%) = (net absorbance of Cx/net absorbance of COV+) × 100(5)

Second, the composite toxicity estimation was assessed in the LF90/10 and LF100 extracts only from fish specimens previously identified as positive or suspect in the qualitative screening step. To this end, eight distinct concentrations (obtained by a serial dilution 1:2) of CTX1B or fish extracts were analyzed to generate a dose-response curve from which the EC_50_ value could be determined. A CTX1B standard solution sourced from ILM ranging from 0.15 to 19.05 pg mL^−1^ (final concentrations) were tested in parallel with each fish sample. Among the suspect and positive specimens, final concentrations ranged from 74 to 9524 ng mL^−1^ for LF90/10 dry extracts (#17, #20, and #22) and from 372 to 47,619 ng mL^−1^ for LF90/10 dry extracts (#1–3 and #18) and for LF100 dry extract (#22). Each concentration was tested in triplicate in OV− and OV+ conditions. Toxicity of each sample was then evaluated with dose-response curves established by plotting net absorbance values vs. toxin standard or dry extract concentrations based on the sigmoidal model 4PL using GraphPad Prism software version 9.0.2. The raw EC_50_ values were expressed in mg of dry extracts mL^−1^ and converted into mg of flesh eq. mL^−1^ based on the “dry extract weight/fresh flesh weight” ratio characterizing each fish sample. Indeed, for the same quantity of 10 g extracted flesh, the quantity of dry extract obtained varied from 1.5 to 20.9 mg for the samples, as also observed in previous studies [52,108]. Thus, the “dry extract weight/fresh flesh weight” ratio, being specific to each sample, was taken into account for the conversion of the EC_50_ values expressed in mg of flesh eq. mL^−1^.

For pure CTX1B (ILM), the mean EC_50_ value was 1.76 ± 0.07 pg mL^−1^ (*n* = 4). The LOD and LOQ of the LF90/10 fractions were determined according to the following formulas:

LOD = (EC_80_ of CTX1B/MCE) (6)

LOQ = (EC_50_ of CTX1B/MCE) (7)

and established at 0.015 ± 0.001 to 0.207 ± 0.012 and 0.026 ± 0.001 to 0.368 ± 0.015 µg CTX1B eq. kg^−1^ flesh, respectively, depending on the “dry extract weight/fresh flesh weight” ratio of fish samples. This variation of LOD and LOQ values according to fish specimens and/or species is well documented in previous studies [52,109].

The composite cytotoxicity in fish extracts was finally calculated by comparing the EC_50_ values of CTX1B and fish extracts determined in the same experiment according to Viallon et al. [52], using the following equation:

Toxin content = (EC_50_ of CTX1B/EC_50_ of fish flesh) (8)

The toxin content was expressed in µg CTX1B eq. kg^−1^ of fish flesh and reported as mean concentration ± SD per fish sample tested in one microplate in four independent assays (*n* = 4) run at different days.

### 3.5. Chemical Methods—Liquid Chromatography Tandem Mass Spectrometry (LC–MS/MS)

For LC–MS/MS analyses, an aliquot of the liposoluble extracts equivalent to 100–200 g of flesh according to fish specimen was prepared (Figure 6) and resuspended in 5 mL of ethyl acetate/methanol (EtOAc/MeOH) 85/15 and purified using two successive solid phase extraction (SPE) clean up steps. First, Bond Elut Florisil^®^ cartridges (500 mg, Agilent technologies, Santa Clara, CA, USA) were conditioned with 3 mL of EtOAc/MeOH 85/15. Then, 2 mL of sample extracts were loaded onto the cartridge and eluted with 2 × 2 mL of the same solvent. The two fractions were combined (1 × 2 mL + 2 × 2 mL) and evaporated under nitrogen (N_2_) at 40 °C. The purified extract was dissolved in MeOH/H_2_O 70/30 (2 mL). For the second SPE purification, Bond Elut LRC C18 cartridges (500 mg, Agilent technologies, Santa Clara, CA, USA) were conditioned with 3 mL of MeOH/H_2_O 70/30, then the purified extract was loaded onto the cartridge. The C18 cartridges were then washed with 3 mL MeOH/H_2_O 75/25, and then CTXs were eluted with 2 × 3 mL of MeOH/H_2_O 90/10. The two eluting fractions (2 × 3 mL) were combined and evaporated under N_2_ at 40 °C. The purified extract was reconstituted with 500 µL of MeOH prior to LC–MS/MS analysis at a concentration ranging from 80 to 160 g flesh eq. mL^−1^. LC–MS/MS analyses were performed using a UHPLC system (UFLC Nexera, SHIMADZU, Japan) coupled to a hybrid triple quadrupole-linear ion-trap API4000 QTRAP mass spectrometer (SCIEX, CA, USA) equipped with a TurboV^®^ electrospray ionization source (ESI). The instrument control, data processing, and analysis were conducted using Analyst software 1.6.3 (Sciex, CA, USA). The quantitative targeted analysis of CTXs was investigated according to the method previously described [103]. The selected *m/z* transitions are listed in Table S3. Calibration solution of CTX1B (ILM, Papeete, Tahiti, French Polynesia) was prepared in MeOH with concentrations ranging from 1 to 10 ng mL^−1^. For fish samples, 5 µL of sample solution was injected corresponding to 400 to 800 mg flesh equivalents. The LOD and LOQ were determined with the ordinary least-squares regression data method [110], at 0.006 and 0.0125 µg CTX1B kg^−1^ of fish tissue, respectively.

### 3.6. Statistical Analyses

No statistical comparison could be made with MBA or LC–MS/MS data, as only one value per specimen was obtained. Conversely, the repeatability of CTX contents obtained with rRBA (*n* = 3) and CBA-N2a (*n* = 4) could be evaluated based on the coefficient of variation (CV) calculated following the formula ((SD/mean) × 100%).

To determine whether rRBA values (*n* = 3) differed significantly with CBA-N2a values (*n* = 4), the Student’s t-test (multiple unpaired) was performed using GraphPad Prism software version 9.0.2 (GraphPad, San Diego, CA, USA).

## 4. Conclusions

This study is the first to provide evidence of the potential contamination of deep-water predatory fishes with significant levels of CTXs in a long-standing CP hotspot of French Polynesia. Despite the limited number of specimens analyzed per species (mainly due to the artisanal nature of the fishing gear), CTX-like activities were detected in seven individuals and one pooled samples of deep-water fish using three biological methods (MBA, rRBA, and CBA-N2a), as well as the presence of three CTX analogs confirmed by LC–MS/MS in three of the toxic specimens. It is also worth mentioning that there are presently very few studies in the bibliography that document the comparison of four CTX detection/quantitation methods in fish, and our results showed good agreement between the methods used, with discrepancies observed only in samples contaminated with low levels of CTXs.

Fortunately for local populations, CP cases linked to the consumption of these highly prized deep-water fish species remain scarce [92]. If more cases were to occur, the present results would undoubtedly benefit from further monitoring efforts and analyses of additional deep-water fish specimens. This novel finding has, however, important implications in terms of ciguatera risk management, in that it stresses the need for a systematic monitoring of CTXs in these deep-sea fish species, most notably in ciguatera hotspots, and in areas where they constitute an important part of the commercial deep-sea fishery, as is the case for *Pristipomoides filamentosus* and *Etelis coruscans* in many countries of the Asia–Pacific region [3,7,9].

## Figures and Tables

**Table 1:** Toxic activity on mice of deep-water fish specimens collected by two local fishermen in 2003 from the Gambier Archipelago using the mouse bioassay (MBA).

Species	Site	Sample	Flesh Equivalent Injected (g)	Survival Time of Mice ^c^	Symptoms	Total Fish Flesh Toxicity (MU g^−1^) ^d^	CTX-like Content (µg CTX1B eq. kg^−1^) ^e^
*Saloptia powelli*	Unknown ^a^	1–3 ^b^	200	≥24 h	No symptoms	<0.005	<LOD ^f^
	Tauna	4–5 ^b^	200			<0.005	<LOD
	East Tepapuri	6	200			<0.005	<LOD
	North Tepapuri	7	200			<0.005	<LOD
	Gaioio	8	200			<0.005	<LOD
	Tenoko	9	200			<0.005	<LOD
	Tekava	10–11 ^b^	200			<0.005	<LOD
	Totegegie	12–13 ^b^	100			<0.01	<LOD
	Gaioio	14–15 ^b^	100			<0.01	<LOD
*Epinephelus tuamotuensis*	Gaioio	16	100	≥24 h	No symptoms	<0.01	<LOD
	Tokorua	17	10	Death after 5 h/death in the night	Diarrhea, dyspnea, cyanosis	0.10	0.70
*Etelis coruscans*	Unknown	18	25	≥24 h/death in the night	Diarrhea	0.04	0.28
	Tokorua	19	200	Death after 5 h/6 h	Diarrhea, dyspnea, flickering walk ataxia, jump before death	0.005	0.04
*Pristipomoides filamentosus*	Unknown	20	50	Death after 3 h 15/4 h 30	Diarrhea, dyspnea, ataxia	0.02	0.14
	Tokorua	21	100	≥24 h	No symptoms	<0.01	<LOD
*Eumegistus illustris*	Tokorua	22	5	Death after 2 h 50/3 h 50	Diarrhea, dyspnea, priapism, cyanosis	0.20	1.40

^a^ NI = no information about the fishing site location. ^b^ Fish samples from the same site and the same date were pooled together. ^c^ Two mice were injected. ^d^ Total fish flesh toxicity is expressed in mouse unit per gram of fish flesh (MU g^−1^). ^e^ CTX-like content was estimated considering that 1 MU is equivalent to 7 ng CTX1B and expressed in µg CTX1B eq. kg^−1^ of fish flesh. ^f^ The limits of detection/quantification LOD = LOQ of the MBA corresponded to 0.035 µg CTX1B eq. kg^−1^ of fish flesh.

**Table 2:** Binding affinity data of deep-water fish specimens collected by two local fishermen in 2003 from the Gambier Archipelago using the radioactive receptor binding assay (rRBA).

Species	Site	Sample	IC_50_^b^	CTX-like Content ^d^
			(mg Flesh eq. mL^−1^)	(µg CTX1B eq. kg^−1^)
*Saloptia powelli*	Unknown ^a^	1	ND ^c^	<LOD ^e^
		2	ND	<LOD
		3	ND	<LOD
	Tauna	4	ND	<LOD
		5	ND	<LOD
	East Tepapuri	6	ND	<LOD
	North Tepapuri	7	ND	<LOD
	Gaioio	8	ND	<LOD
	Tenoko	9	1260 ± 245	0.21 ± 0.04
	Tekava	10	ND	<LOD
		11	ND	<LOD
	Totegegie	12	ND	<LOD
		13	ND	<LOD
	Gaioio	14	ND	<LOD
		15	ND	<LOD
*Epinephelus tuamotuensis*	Gaioio	16	ND	<LOD
	Tokorua	17	435 ± 36.3	0.60 ± 0.05
*Etelis coruscans*	Unknown	18	967 ± 117	0.27 ± 0.03
	Tokorua	19	384 ± 121	0.72 ± 0.21
*Pristipomoides filamentosus*	Unknown	20	1076 ± 215	0.25 ± 0.06
	Tokorua	21	473 ± 12.7	0.55 ± 0.01
*Eumegistus illustris*	Tokorua	22	231 ± 45.0	1.15 ± 0.22

^a^ Unknown = no information about the fishing site location. ^b^ Each value represents the mean ± SD of three aliquots per sample tested once. ^c^ ND = no affinity detected. ^d^ CTX-like content was calculated according to Equation (3) (Section 3.4.1). ^e^ LOD: the limit of detection for fish samples was estimated at 0.07 ± 0.02 µg CTX1B eq. kg^−1^ of fish flesh.

**Table 3:** Composite cytotoxicity data of deep-water fish specimens collected by two local fishermen in 2003 from the Gambier Archipelago using the neuroblastoma cell-based assay (CBA-N2a).

Species	Site	Sample	Viability Percentages ^c^	EC_50_^e^ ng Dry Extract mL^−1^	EC_50_^e^ mg Flesh eq. mL^−1^	CTX-like Content (µg CTX1B eq. kg^−1^)
*Saloptia powelli*	Unknown ^a^	1–3 ^b^	53.5%	9139 ± 652	17.9 ± 1.28	0.10 ± 0.01 ^b^
	Est Tepapuri	6	102.8%	ND ^f^	ND ^f^	<LOD ^h^
	North Tepapuri	7	94.0%	ND	ND	<LOD
	Gaioio	8	100.5%	ND	ND	<LOD
	Tenoko	9	116.4%	ND	ND	<LOD
	Tekava	10–11 ^b^	116.4%	ND	ND	<LOD
*Epinephelus tuamotuensis*	Tokorua	17	1.1%	1391 ± 91	0.67 ± 0.04	2.68 ± 0.14
*Etelis coruscans*	Unknown	18	5.7%	4494 ± 444	2.39 ± 0.24	0.74 ± 0.04
*Pristipomoides filamentosus*	Unknown	20	1.3%	1080 ± 136	7.20 ± 0.91	0.25 ± 0.02
*Eumegistus illustris*	Tokorua	22	0.7%	818 ± 109	0.63 ± 0.08	2.83 ± 0.23
(LF100)			65.3% ^d^	19,924 ± 1728 ^g^	15.3 ± 1.33	0.12 ± 0.01

^a^ Unknown = no information about the fishing site location. ^b^ Fish samples from the same site were pooled together. ^c^ Screening results of LF90/10 extracts are expressed in N2a cell viability percentages (Section 3.4.3). ^d^ Screening result of LF100 extracts from the *E. illustris* specimen only was presented as percentage value was <80%. ^e^ Each value represents the mean ± SD of three independent experiments. ^f^ ND = no cytotoxicity detected. ^g^ The maximum concentration of LF90/10 and LF100 extracts for matrix interference were 10,000 and 100,000 pg µL^−1^ of dry extract, respectively [53]. ^h^ LOD: the limit of detection was estimated at 0.015 ± 0.001 to 0.207 ± 0.012 µg CTX1B eq. kg^−1^ of flesh according to the dry extract/flesh weight ratio of samples.

**Table 4:** The liquid chromatography tandem mass spectrometry (LC–MS/MS) estimation of the relative concentrations of Pacific CTXs congeners in deep-water fish collected by two local fishermen in 2003 in the Gambier Archipelago using CTX1B (ILM) calibration curve.

Fish Species	Site	Sample	CTX Content per Analog (µg CTX1B kg^−1^)			Total CTX Content
			CTX1B	52-*epi*-54-deoxyCTX1B	54-deoxyCTX1B	(µg kg^−1^)
*Saloptia powelli*	Unknown ^a^	1–3 ^b^	<LOD ^c^	<LOD	<LOD	<LOD
	Tekava	10–11 ^b^	<LOD	<LOD	<LOD	<LOD
*Epinephelus tuamotuensis*	Tokorua	17	0.28	<LOD	<LOD	0.28
*Etelis coruscans*	Unknown	18	<LOD	<LOD	<LOD	<LOD
*Pristipomoides filamentosus*	Unknown	20	0.08	<LOD	<LOD	0.08
*Eumegistus illustris*	Tokorua	22	0.36	0.49	0.26	1.11

^a^ Unknown = no information about the fishing site location. ^b^ Fish from the same site were pooled together. ^c^ LOD: the detection limit was determined at 0.006 µg CTX1B kg^−1^ of fish flesh.

**Table 5:** Summary of ciguatoxin levels in deep-water fish samples collected by two local fishermen in 2003 from the Gambier Archipelago, as assessed by the radioactive receptor biding assay (rRBA), the mouse bioassay (MBA), the neuroblastoma cell-based assay (CBA-N2a) (expressed in µg CTX1B eq. kg^−1^ of flesh), and liquid chromatography tandem mass spectrometry (LC–MS/MS) (expressed in µg CTX1B kg^−1^ of flesh).

Species	Site	Sample	rRBA	MBA	CBA-N2a	LC–MS/MS
*Saloptia powelli*	Unknown ^a^	1	<LOD	<LOD ^b^	0.10 ± 0.01 ^b^	<LOD ^b^
		2	<LOD			
		3	<LOD			
	Tauna	4	<LOD	<LOD		
		5	<LOD			
	East Tepapuri	6	<LOD	<LOD	<LOD	
	North Tepapuri	7	<LOD	<LOD	<LOD	
	Gaioio	8	<LOD	<LOD	<LOD	
	Tenoko	9	0.21 ± 0.04	<LOD	<LOD	
	Tekava	10	<LOD	<LOD ^b^	<LOD ^b^	<LOD ^b^
		11	<LOD			
	Totegegie	12	<LOD	<LOD ^b^		
		13	<LOD			
	Gaioio	14	<LOD	<LOD ^b^		
		15	<LOD			
*Epinephelus tuamotuensis*	Gaioio	16	<LOD	<LOD		<LOD
	Tokorua	17	0.60 ± 0.05	0.7	2.68 ± 0.14	0.28
*Etelis coruscans*	Unknown	18	0.27 ± 0.03	0.28	0.74 ± 0.04	<LOD
	Tokorua	19	0.72 ± 0.21	0.04		
*Pristipomoides filamentosus*	Unknown	20	0.25 ± 0.06	0.14	0.25 ± 0.02	0.08
	Tokorua	21	0.55 ± 0.01	<LOD		
*Eumegistus illustris*	Tokorua	22	1.15 ± 0.22	1.4	2.83 ± 0.23	1.11
(LF100 ^c^)					0.12 ± 0.01	

^a^ Unknown: no information about the fishing site location. ^b^ Fish samples from the same site were pooled for MBA, CBA-N2a, and LC–MS/MS analyses. ^c^ LF100: only the fraction of specimen #22 could be quantified. The limits of detection/quantification (LOD/LOQ) of the MBA were defined as 0.035 µg CTX1B eq. kg^−1^ of fish flesh. The limits of detection (LOD) for fish samples were estimated at 0.07 ± 0.02 µg CTX1B eq. kg^−1^ and 0.015 ± 0.001 to 0.207 ± 0.012 µg CTX1B eq. kg^−1^ of flesh for rRBA and CBA-N2a, respectively, and 0.006 µg CTX1B kg^−1^ of flesh for LC–MS/MS. The limits of quantification (LOQ) for fish samples were estimated at 0.13 ± 0.01 and 0.026 ± 0.001 to 0.368 ± 0.015 µg CTX1B eq. kg^−1^ of flesh for rRBA and CBA-N2a respectively, and 0.0125 µg CTX1B kg^−1^ of flesh for LC–MS/MS.

## Data Availability

Not applicable.

## Supplementary Materials

The following are available online at https://www.mdpi.com/article/10.3390/md19110644/s1, Table S1: Ciguatera poisonings caused by the consumption of deep-water fish in French Polynesia from 1999 to 2021, Table S2: List of deep-water fish specimens collected by two local commercial fishermen in 2003 from the Gambier Archipelago (French Polynesia), Table S3: Selected *m*/*z* transitions and liquid chromatography tandem mass spectrometry (LC–MS/MS) instrument parameters used for the scheduled MRM method on C18 Zorbax Eclipse plus column (50 × 2.1 mm, Agilent technologies) with a linear gradient of Eluent A (water) and eluent B (methanol), both eluents containing 2 mM ammonium formiate and 50 mM formic acid, Figure S1: Pictures of deep-water fish species collected and send by fishermen from the Gambier Archipelago in 2003 analyzed in the present study: (a) *Etelis coruscans*, (b) *Pristipomoides filamentosus*, (c) *Saloptia powelli*, (d) *Epinephelus tuamotuensis*, and (e) *Eumegistus illustris*.
