# Supplementary material for: Deep-Water Fish Are Potential Vectors of Ciguatera Poisoning in the Gambier Islands, French Polynesia
Source: Mar Drugs. 2021 Nov 17;19(11):644. doi: 10.3390/md19110644 (PMC8621427; doi:10.3390/md19110644)
Supplement: Supplementary file 1 [file marinedrugs-19-00644-s001.zip › marinedrugs-1439566-supplementary-update.pdf]

marine drugs

MDPI

Supplementary Materials: Deep-Water Fish are Potential Vectors of Ciguatera Poisoning in the Gambier Islands, French Polynesia

Hélène Taiana Darius [1,*], Taina Revel [1], Philippe Cruchet [1], Jérôme Viallon [1], Clémence Mahana iti Gatti [1], Manoëlla Sibat [2], Philipp Hess [2] and Mireille Chinain [1]

[1] Laboratory of Marine Biotoxins, Institut Louis Malardé (ILM), UMR 241-EIO (IFREMER, ILM, IRD, University of French Polynesia), PO Box 30, 98713 Papeete, Tahiti, French Polynesia; trevel@ilm.pf (T.R.); pcruchet@ilm.pf (P.C.); jviallon@ilm.pf (J.V.); cgatti@ilm.pf (C.M.i.G); mchinain@ilm.pf (M.C.)
[2] Laboratoire Phycotoxines, DYNECO, Institut Français de Recherche pour l'Exploitation de la MER, F-44000 Nantes, France; manoella.sibat@ifremer.fr (M.S.); philipp.hess@ifremer.fr(P.H.)
* Correspondence: tdarius@ilm.pf; Tel.: +689-40-416-484

Table S1. Ciguatera poisonings caused by the consumption of deep-water fish in French Polynesia from 1999 to 2021.

Event	Number of patient	Year of event	Location	Fish name
#1*	1	1999	Anaa, Tumaotu	« Paru », Jobfish
#2*	3	1999	Unknown	« Paru », Jobfish
#3	1	2003	Mangareva, Gambier	« Uravena », Oilfish (*Ruvettus pretiosus*)
#4	1	2003	Mangareva, Gambier	« Paru », Jobfish
#5	2	2003	Unknown	« Paru », Jobfish
#6	1	2006	Rangiroa, Tuamotu	« Paru », Jobfish
#7*	1	2006	Raroia, Tuamotu	« Paru », Jobfish
8*	1	2010	Rurutu,Australes	« Uravena », Oilfish (*Ruvettus pretiosus*)
#9*	6	2010	Tubuai,Australes	« Uravena », Oilfish (*Ruvettus pretiosus*)
#10*	2	2011	Rurutu, Australes	« Paru », Jobfish
#11*	1	2011	Hao, Tuamotu	« Paru Tahakari », Rusty jobfish (*Aphareus rutilans*)
#12*	1	2020	Mangareva, Gambier	« Paru », Jobfish

*Clinical information were reported for eight of these events: diarrhea (70%), vomiting (50%) and nausea(20%), among digestive manifestations; severe hypotension (20%) and bradycardia (20%) among cardiovascular manifestations, then cold allodynia(70%), tingling (60%), muscular disorders (70%), arthralgia (60%), itching (50%), dizziness/balance disorder (50%), paresthesia (40%), headache (30%), vision disorders (30%), dysgeusia (20%) and throat and mouth burning sensation (20%) among neurological and general manifestations. According to the patients, symptoms appeared within 12h after the consumption of the fish, 2h for the earliest. Note that both the clinical manifestations and the incubation time are compatible with CP diagnosis.

Table S2. List of deep-water fish specimens collected by two local commercial fishermen in 2003 from the Gambier Archipelago (French Polynesia).

Family	Species	Common name	Tahitian name	Diet[a]	Site	Date	Sample #	Depth (m)	Weight (g)	Length (cm)
Serranidae	*Saloptia powelli* (Smith, 1964)	Golden grouper	Paru hoa	Finfishes, Crustaceans	Unknown[b]	23/10/03	1	NI	NI	NI
							2	NI	NI	NI
							3	NI	NI	NI
					Tauna	17/12/03	4	300	698	38
							5	300	452	34
					East Tepapuri	18/12/03	6	350	707	38.5
					North Tepapuri	19/12/03	7	300	967	42.5
					Gaioio	20/12/03	8	300	729	38.5
					Tenoko	21/12/03	9	250-300	927	42
					Tekava	22/12/03	10	300	963	42
						23/12/03	11	280-350	789	39
					Totegegie	25/12/03	12	250	941	42
						26/12/03	13	250	722	37
					Gaioio	27/12/03	14	250	928	39
						28/12/03	15	250	740	38
	Epinephelus tuamotuensis (Fourmanoir, 1971)	Reticulate grouper	Hapu'u hei	Finfishes, crustaceans, small « eels »	Gaioio	28/11/03	16	250-300	2426	NI
					Tokorua	11/12/03	17	300	5000	68
Lutjanidae	*Etelis coruscans* (Valenciennes, 1862)	Deepwater longtail red snapper	Paru i'ihi Onaga	Cephalopods, finfishes, benthic crustaceans	Unknown	23/10/03	18	NI	2100	NI
					Tokorua	11/12/03	19	300	2340	58
	Pristipomoides filamentosus (Valenciennes, 1830)	Crimson jobfish	Paru utu	Cephalopods, finfishes, benthic crustaceans echinoderms, sponges	Unknown	24/11/03	20	350	3549	NI
					Tokorua	11/12/03	21	300	2940	58
Bramidae	*Eumegistus illustris* (Jordan & Jordan, 1922)	Brilliant pomfret	Paru papio	Cephalopods, finishes micronekton, crustaceans	Tokorua	11/12/03	22	300	*5000*[c]	*45*[c]

[a]Diet information collected from Ponsonnet (2002), Gray (2016), and www.fishbase.org (2021). [b]Unknown = no information about fishing site, depth, weight, or size data for these fish samples. [c]Weight and size were indicated for a portion of the fish, the whole fish was not provided.

Table S3. Selected *m/z* transitions and liquid chromatography tandem mass spectrometry (LC-MS/MS) instrument parameters used for the scheduled MRM method on C18 Zorbax Eclipse plus column (50*2.1 mm, Agilent technologies) with a linear gradient of Eluent A (water) and eluent B (methanol), both eluents containing 2 mM ammonium formiate and 50 mM formic acid.

**Compound**	**Detection window** (min)	**Precursor ion** (Q1) *m/z*	**Product ion** (Q3) *m/z*	**DP** (eV)	**CE** (eV)	**CXP** (eV)
CTX1B, CTX1A	3.1 ± 1	1128.6 $[M+NH_4]^+$	1093.6	105	20	12
			1075.6	105	30	12
			95.1	105	90	20
M-*seco*-CTX3C	4.7 ± 1	1041.6 $[M+H]^+$	1023.6	105	30	12
			1005.6	105	20	12
			125.1	105	50	18
2,3-dihydro-2-hydroxyCTX3C and 2,3-dihydro-3-hydroxyCTX3C	5.4 ± 1	1058.6 $[M+NH_4]^+$	1023.6	105	30	12
			1005.6	105	20	12
			125.1	105	50	18
2,3-dihydro-2,3-dihydroxyCTX3C	6.0 ± 1	1074.6 $[M+NH_4]^+$	1039.6	105	30	12
		1057.6 $[M+H]^+$	1039.6	105	20	12
			125.1	105	50	18
51-hydroxyCTX3C	6.3 ± 1	1056.6 $[M+NH_4]^+$	1021.6	105	30	12
		1039.6 $[M+H]^+$	1021.6	105	20	12
			1003.6	105	20	12
M-*seco*-CTX4A/4B	6.5 ± 1	1096.6 $[M+NH_4]^+$	1043.7	105	30	12
		1079.6 $[M+H]^+$	1043.7	105	20	12
			125.1	105	50	18
52-*epi*-54-deoxyCTX1B and 54-deoxyCTX1B	6.8 ± 1	1112.6 $[M+NH_4]^+$	1077.6	105	20	12
			1059.6	105	30	12
			95.1	105	90	20
CTX3C isomers (1), (2) and (3)	7.6 ± 1	1040.6 $[M+NH_4]^+$	1005.6	105	30	12
		1023.6 $[M+H]^+$	1005.6	105	20	12
			125.1	105	20	12
CTX3C, 49-*epi*CTX3C (CTX3B) and isomer (4)	10.5 ± 1	1040.6 $[M+NH_4]^+$	1005.6	105	30	12
		1023.6 $[M+H]^+$	1005.6	105	20	12
			125.1	105	50	18
CTX4A and CTX4B	12.2 ± 1	1078.6 $[M+NH_4]^+$	1043.6	105	30	12
		1061.6 $[M+H]^+$	1043.6	105	20	12
			125.1	105	50	18

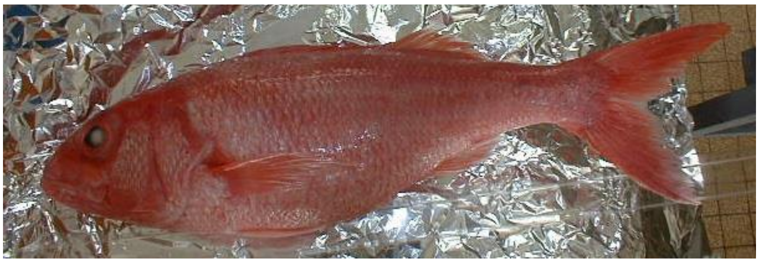

(a)

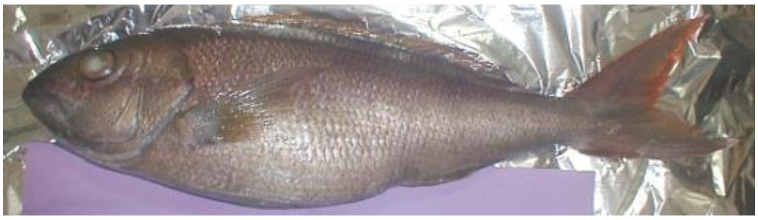

(b)

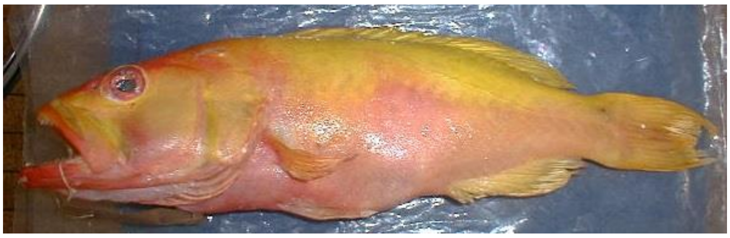

(c)

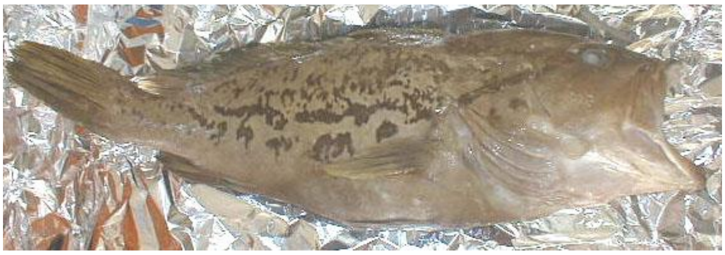

(d)

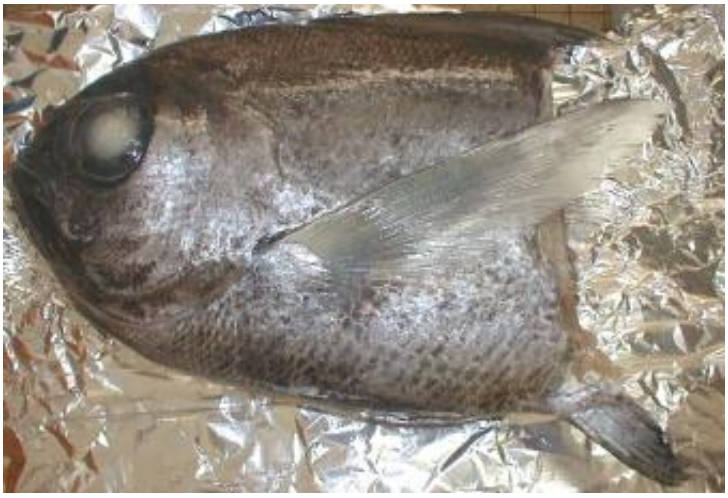

(e)

Figure S1. Pictures of deep-water fish species collected and send by two local commercial fishermen from the Gambier Archipelago in 2003 analyzed in the present study: (a) *Etelis coruscans*, (b) *Pristipomoides filamentosus*, (c) *Saloptia powelli*, (d) *Epinephelus tuamotuensis*, and (e) *Eumegistus illustris*.
